# Corporate Characteristics and Adoption of Good Manufacturing Practice for Dietary Supplements in Japan

**DOI:** 10.3390/ijerph17134748

**Published:** 2020-07-01

**Authors:** Keigo Sato, Kota Kodama, Shintaro Sengoku

**Affiliations:** 1 Department of Innovation Science, School of Environment and Society, Tokyo Institute of Technology, Tokyo 108-0023, Japan; sato.k.bz@m.titech.ac.jp; 2 Graduate School of Technology Management, Ritsumeikan University, Osaka 567-8570, Japan; kkodama@fc.ritsumei.ac.jp; 3 Life Style by Design Research Unit, Institute for Future Initiatives, University of Tokyo, Tokyo 113-0033, Japan

**Keywords:** functional food, quality certification, industry convergence, organizational capability, GMP, good manufacturing practices

## Abstract

Good manufacturing practice (GMP) is advocated and implemented as a standardized procedure for manufacturing dietary supplements. However, in Japan as a case, only half of the manufacturers in this field so far adopt it. To address this issue, the present study aims to explore the effect of key characteristics of a company on the adoption of and compliance with GMP for dietary supplements. The focus is on the effect of expertise in the pharmaceutical industry. The relationships between company characteristics and the adoption of GMP were analyzed for 90 manufacturers in the dietary supplement industry in Japan. A binomial logistic regression analysis showed that each of the following three factors had a positive and significant effect on the company’s adoption of GMP: company size in terms of revenue (odds ratio = 1.04, *p* = 0.019), possession of a manufacturing license for pharmaceutical products (13.7, *p* = 0.003), and number of own product categories manufactured (3.93, *p* = 0.00009). These findings strongly suggest that the company’s manufacturing capability of pharmaceutical products works as a key driver for the better adoption of a quality standard in the dietary supplement industry in Japan. Few considerations were made for conditions of the adoption and implementation of GMP. The present study empirically contributes by providing key clues for issues in the dietary supplement industry and by forming a theoretical base for policymakers and the regulatory authorities.

## 1. Introduction

A dietary supplement is a food shaped as a soft capsule, hard capsule, tablet, granule, or liquid that differs from regular food and contains functional physiological ingredients. It is expected to contribute to promoting health and preventing disease in aging societies, and it is considered a way of self-medication with the potential to reduce the public health burden. The market for dietary supplements expanded steadily in accordance with expectations and increased food technology innovation [1,2,3]. The dietary supplement industry incorporated various business entities: pharmaceutical and food companies, small and medium-sized enterprises (SMEs), start-up companies that use novel functional ingredients and/or local specialty foods, new entrants from other industries, and original equipment manufacturers (OEMs). 

**Figure 1:**
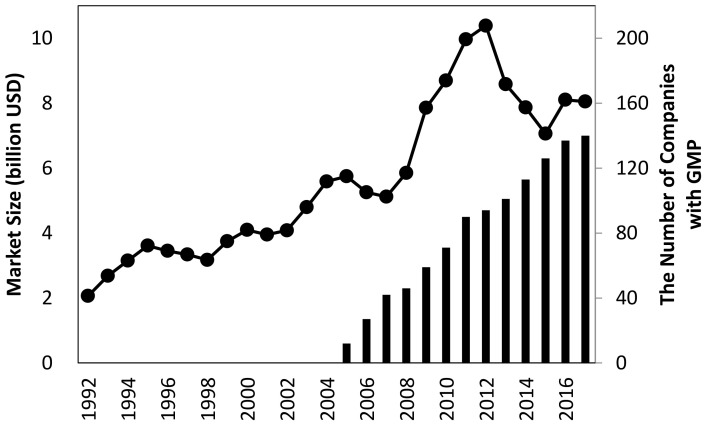
Historical transition of the market size of dietary supplements (the line) and number of good manufacturing practice (GMP)-licensed manufacturers for dietary supplements in Japan (the bars). Data on the market size and the number of companies were obtained from commercially available sources [4] and from GMP certification organizations (Japan Health and Nutrition Food Association—JANFA, and Japanese Institute for Health Food Standards—JIHFS).

In Japan, the size of the dietary supplement market is growing and currently totals $8 billion United States dollars (USD) (Figure 1), and the industrial ecosystem is developed based on the transdisciplinary environment described above. Notably, more than 70% of dietary supplement manufacturers outsource their manufacturing process to OEMs.

**Figure 2:**
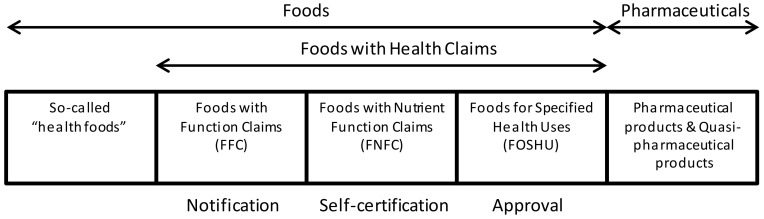
Categories ranging from food to pharmaceuticals containing Foods with Health Claims.

As a recent regulatory movement, a unique Food with Function Claims (FFC) system was launched in Japan in 2015. This system was introduced with the policy intention to use functional food and dietary supplements for health promotion and disease prevention, as well as for control of medical expenses. FFC are foods submitted to the Secretary General of the Consumer Affairs Agency (CAA) as products whose labels bear function claims based on scientific evidence, which is the responsibility of food business operators [5]. Before the launch of the system, making function claims on food labels was only allowed for government-approved Foods for Specified Health Uses (FOSHU) and self-certified Foods with Nutrient Function Claims (FNFC) that complied with the specifications and standards designated by the government. The system of Foods with Health Claims (FHC) can be delineated into three categories, as shown in Figure 2.

In the current FFC system, the government does not evaluate the safety and effectiveness of function claims. Food business operators submit an appropriate function claim based on scientific evidence for which they are responsible. Scientific evidence for function claims must be obtained through a clinical trial or systematic review of the literature. To propose function claims under the FFC system, it is required to submit a premarket notification and to label the package in accordance with the Food Labeling Standards pursuant to the Food Labeling Act, as well as the “Guidelines on Notification of Foods with Function Claims”. This system provides more information about functional food products to consumers and helps especially small companies develop functional foods [6,7,8]. The FFC system accelerates new entrants and the further growth of the market.

Alongside the growth of the market and industry, quality issues such as inappropriate manufacturing process management and insufficient ingredient amounts emerged [9]. According to the Food and Drug Administration (FDA) report about dietary supplements in 2013, 6307 adverse event reports from 2008 to 2011 resulted in serious outcomes—unspecified important medical events of a serious nature (53%), hospitalization (29%), serious injuries or illnesses (20%), resulted in a life-threatening condition (8%), and death (2%) [10]. In Japan, the Ministry of Health, Labor, and Welfare (MHLW) drew attention to quality issues by presenting examples of health hazards caused by health foods [11]. The factors that affect the quality of the product are the quality of the raw materials themselves, the addition of multiple materials, and impurities. Since raw materials are not standardized, purity and ingredient amounts vary from manufacturer to manufacturer. In the case of natural plant extract ingredients, in addition to the fact that the ingredients contained are often not specified, the ingredients contained in the product vary depending on where it is grown and when it is harvested. The National Institute of Health and Nutrition disseminates information on safety and health damage for health foods through their website [12].

It is problematic that consumers can hardly determine whether a product is sufficiently qualified when purchasing it, since all products have a similar shape and appearance, for example, tablets or capsules. Therefore, quality assurance standards were developed and implemented to reduce this asymmetry of information between manufacturers and consumers. For example, in the pharmaceutical industry, good manufacturing practice (GMP) was adopted as a means of quality assurance and control. This regulatory standard ensures the compliance of pharmaceutical manufacturers with defined manufacturing and packaging procedures. 

Several studies reported that the implementation of GMP by a company effectively induces profound behavioral changes in the organization and individuals and, furthermore, enhances the level of knowledge and awareness of the significance of product quality and safety [13]. GMP is used to manage an appropriate operation under a manufacturing process in accordance with defined standards to ensure that quality control is properly implemented. Quality risks such as misidentification or mislabeling of ingredients, adulterations, substitutions, and contaminations in the production process can be decreased through the GMP system, ultimately decreasing the number of consumer complaints [14]. In addition, the implementation of GMP reportedly improves the working environment and promotes employee motivation and productivity [15]. The International Conference on Harmonization (ICH) provides current good manufacturing practice (cGMP).

GMP is advocated as a standardized regulatory system for the manufacturing process, as in the pharmaceutical industry [3,15]. Some countries including the United States, European Union (EU), Korea, Taiwan, and China already adopt GMP as a regulatory requirement for product qualification [3,7,16,17]. However, in Japan, GMP for dietary supplements is still voluntary. In Japan, two organizations certify the GMP for dietary supplements, namely, the Japan Health and Nutrition Food Association (JANFA) and the Japanese Institute for Health Food Standards (JIHFS). However, since 2005, only around 150 Japanese OEM companies (half of all OEM companies) adopted GMP (Figure 1). The GMP system is not mandated, because dietary supplements are not legally defined in Japan [18].

The present study aims to explore the effect of the key characteristics of a company on the adoption of and compliance with GMP for dietary supplements, with a focus on the effect of expertise in the pharmaceutical industry. This study also explores how to successfully implement GMP to further innovate dietary supplements by identifying factors that influence the adoption (or rejection) of GMP. Furthermore, the required organizational capability needed for implementation is discussed.

From the perspective of product characteristics and institutional aspects, dietary supplements are positioned between food and medicine in the regulatory spectrum [19]. In this regard, several studies on regulatory science and innovation management discussed the influence of technological and regulatory trajectories and the path-dependent or creative mechanism of the dietary supplement industry.

The dietary supplement industry is an emerging one formed by the industrial integration of the pharmaceutical and food industries [20]. The concept of industrial convergence highlights that new industry sectors form as knowledge, technology, and institutions from various industries fuse [21,22]. In the case of dietary supplements, the pharmaceutical industry introduced technical competences, whereas the food industry focused on marketing competence [23].

Several studies revealed the significance of implementing standards for quality control in the manufacturing process, such as pursuing operational excellence in improving product quality and productivity, as well as educational contributions to employees. Others are external effects such as building healthy relationships with customers and the positive effects of product marketing [24,25,26]. Reportedly, the size of an enterprise affects the adoption of quality control standards. Notably, small-sized companies with relatively undifferentiated product lines tend to be motivated to adopt a quality control standard for differentiation purposes in the competitive marketplace and as a requirement by customers with relatively large buying power [25].

As a negative impact, additional costs for quality assurance, including human resource development or hiring and investment for GMP-complying facilities and equipment, are obstacles to the introduction of GMP [27,28]. Therefore, larger enterprises tend to more proactively introduce quality standards, because of a relatively lower cost burden than small and mid-sized enterprises [29].

As discussed, prior studies revealed the existence of external and internal factors that affect the adoption and implementation of quality control standards. In the business of contracted manufacturing organizations (CMOs) for dietary supplements, the cost burden and organizational capability are considered key internal factors that affect the adoption and implementation of GMP for dietary supplements. Specifically, the introduction and maintenance of GMP-complying manufacturing facilities and equipment for inspection are key elements of direct costs, and building organizational capabilities such as improving operations and employee education may increase indirect costs. These additional costs are considered relatively lighter for larger enterprises in terms of the economy of scale, resulting in the smoother adoption of GMP.

**Hypothesis** **1**: *The adoption rate of GMP for dietary supplements is correlated with the size of the CMO*.

Regarding CMOs in the pharmaceutical sector, their organizational capability to manufacture pharmaceutical products may contribute to dietary supplements, since they operate GMP-level manufacturing processes and quality controls that are mandatory for pharmaceutical products. Their policy, expertise, and human resources could contribute to improving the level of manufacturing process and quality control in the dietary supplement sector, promoting the more proactive adoption of GMP for dietary supplements.

In contrast, as the path-dependency theory points out, non-pharmaceutical manufacturing companies such as food manufacturers have a lower awareness and absorptive capability to adopt GMP for dietary supplements, i.e., they may need to spend additional time and cost to acquire and nurture their organizational capability [30]. Based on these considerations, we hypothesize that barriers to adopting GMP may differ between pharmaceutical and food manufacturers.

**Hypothesis** **2**: *The adoption rate of GMP for dietary supplements is higher among manufacturers of pharmaceutical products than among food product manufacturers*.

Customer relationships are considered key external factors in the adoption of GMP. Manufacturing under the GMP system standardizes and guarantees a certain level of product quality and reduces the level of uncertainty on the customer side. It is also expected to reduce transaction costs between a CMO as an OEM and their clients as end-product manufacturers. Furthermore, in an environment wherein information between OEMs and their clients is asymmetrical, acquiring GMP provides clients with a certificate of quality control in manufacturing, which may contribute to building credibility. In other words, GMP as a certification has a signaling effect that eliminates asymmetrical information and strengthens the relationship between two manufacturers.

In the present study, considering difficulties in measuring the level of a specific customer relationship, the number of product categories that the CMO can manufacture was employed as a proxy for the strength of the customer relationship. Typical categories of dietary supplements are soft capsule, hard capsule, tablet, granule, and liquid. CMOs capable of manufacturing various forms of dietary supplements are considered to sufficiently meet their customers’ requirements. Therefore, the number of categories of manufactured dietary supplements at a CMO was set as a surrogate variable for the strength of the customer relationship.

**Hypothesis** **3**: *The adoption rate of GMP for dietary supplements is correlated with number of product categories a contract manufacturing enterprise can manufacture*.

## 2. Materials and Methods 

Data were collected for 90 OEMs in the Japanese dietary supplement industry in 2016 (the contract manufacturing company guidebook, UBM media; company information database Orbis, Bureau van Dijk). 

We considered the possession of one or more of four types of licenses related to marketing or manufacturing for pharmaceutical or quasi-pharmaceutical products as a surrogate variable for the manufacturing capability of pharmaceutical products. We also considered the possession of one or more of 22 types of licenses related to manufacturing processed food as a surrogate variable for the manufacturing capability of food products. The shape of the dietary supplement was classified into five categories (i.e., soft capsule, hard capsule, tablet, granule, and liquid). The number of product categories ranged from zero (with only a packaging process) to five (with manufacturing processes for all categories mentioned). 

Statistical analyses were performed using R statistical software (version 3.4.1, The R Foundation, Vienna, Austria, 2017). This article does not contain any studies with human or animal subjects performed by any of the authors.

## 3. Results

### 3.1. Descriptive Satistics

Table 1 provides the descriptive statistics of the sample data. The standard deviations for revenue and the number of employees were relatively large, suggesting a considerable difference in the size of these CMOs.

### 3.2. Effect of Company Size

**Figure 3:**
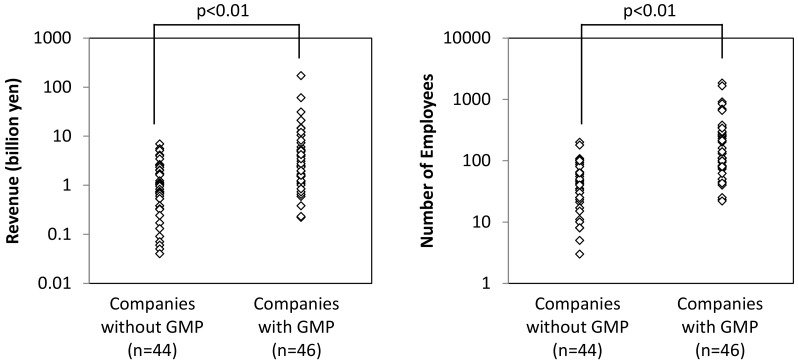
Revenue and number of employees of contracted manufacturing organizations (CMOs) for dietary supplements with and without GMP in Japan.

Figure 3 compares the revenue and number of employees of CMOs with and without GMP (*n* = 46 and 44, respectively). To test hypothesis 1, the Mann–Whitney U test was employed to compare the revenue and number of employees of companies that do or do not comply with GMP for dietary supplements. The test revealed that companies with GMP have significantly more revenue and a larger number of employees than those without it (*p* < 0.01 and 0.01, respectively). This supports hypothesis 1.

### 3.3. Effect of Business Domains

**Figure 4:**
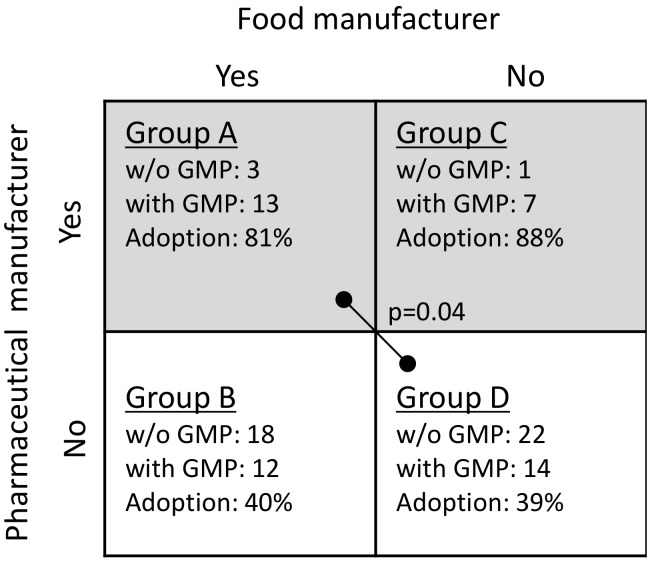
Summary of adoption rates of GMP for dietary supplements for possession of license(s) for manufacturing food and/or pharmaceutical products.

To test hypothesis 2, the samples were divided into four subgroups based on the possession of license(s) for both manufacturing food and pharmaceutical products (subgroup A), only for food (B), only for pharmaceutical (C), or neither (D) (Figure 4). Fisher’s exact test revealed that the adoption rates of GMP varied across these subgroups (*p* < 0.01). Furthermore, we examined the difference between subgroups A (both food and pharmaceutical products) and D (neither) using a *t*-test (Holm method). The results in Figure 4 show that companies with a license for manufacturing pharmaceutical products tend to have a significantly higher adoption rate of GMP for dietary supplements. These results support hypothesis 2.

### 3.4. Effect of Number of Product Categories

**Figure 5:**
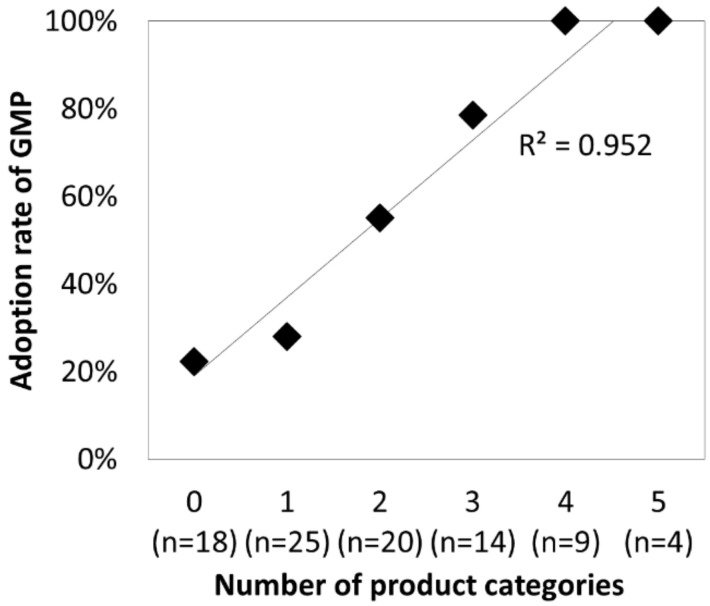
Relationship between the number of product categories and adoption rate of GMP.

To test hypothesis 3 regarding the effect of the number of product categories, the company samples were divided into six subgroups according to the number of categories of manufactured dietary supplement products, ranging from G-0 (no product, i.e., only packaging process) to G-5 (five categories, i.e., soft capsule, hard capsule, tablet, granule, and liquid). Fisher’s exact test revealed that the adoption rates of GMP for dietary supplements varied across subgroups (*p* < 0.00001). Then, the differences were further examined between subgroups G-0 and G-3, G-0 and G-4, G-1 and G-3, and G-1 and G-4 using a *t*-test (Holm method). Figure 5 indicates that the adoption rates of GMP for dietary supplements were significantly correlated with number of product categories manufactured by the companies (*r^2^* = 0.952). This supports hypothesis 3. 

### 3.5. Binomial Logistic Regression Analysis

Based on the above-mentioned results, a binomial logistic regression analysis of the adoption rate of GMP for dietary supplements was conducted. Table 2 shows the correlation coefficients of selected variables: revenue size, possession of a license for manufacturing pharmaceutical products, and the number of categories of manufactured dietary supplement products. Considering multicollinearity caused by the high correlation between revenue and the number of employees (correlation coefficient = 0.82), a model including revenue without the number of employees was run based on the lower value of Akaike’s information criterion (AIC).

Table 3 provides the results of a binominal regression analysis of the adoption rate of GMP for dietary supplements with four independent variables. The odds ratio for revenue size was 1.04 (95% confidence interval 1.01 to 1.09, *p* = 0.019), that for the pharmaceuticals dummy was 13.7 (95% confidence interval 2.85 to 90.8, *p* = 0.003), and that for the number of product categories was 3.93 (95% confidence interval 2.14 to 8.61, *p* = 0.00009). This model confirms the three hypotheses of the study and provides a consolidated view of the contribution of these factors to the adoption of GMP for dietary supplements. 

## 4. Discussion

The results obtained in this study suggested that the size of the company in terms of revenue or the number of employees, license for manufacturing pharmaceutical products, and the number of categories of manufactured dietary supplements contributed to the adoption of GMP for dietary supplements. From a historical viewpoint, the dietary supplement industry emerged through the convergence of the food and pharmaceutical industries, and it was nurtured by these two streams of industrial and organizational capabilities. The present study found that pharmaceutical capability influences a quality level for manufacturing dietary supplements, accompanied by expertise in manufacturing a wide range of dietary supplement products.

In the industry convergence model, multiple convergence steps (knowledge convergence, technology convergence, applicational convergence, and industry convergence) were proposed as a route to industry convergence [23]. Applicational convergence was pointed out as being influenced by the institution and customer needs. The results of this research suggested that, under the application of quality standards such as GMP, it is relatively easy to integrate the pharmaceutical industry into the food industry, whereas it is not so for the food industry into the pharmaceutical industry, i.e., potential asymmetric process/dynamics of the industry convergence, which is expected to provide practical implications to revisiting regulatory conditions in Japan.

Since the introduction of GMP is voluntary in Japan, companies are not uniformly affected by the institution. In the paragraph below, we discuss the reason why Japan’s GMP system is voluntary in comparison with the United States case.

In the United States, GMP for pharmaceuticals was formalized in 1962 [14]. Until 1994, dietary supplements were treated in the same way as regular foods. Since 1994, dietary supplements are regulated under the Dietary Supplement Health Education Act (DSHEA). Dietary supplements are defined as foods with a shape that was processed for consumption, such as a capsule or tablet. In 2007, the cGMPs for dietary supplements were published by the Food and Drug Administration (FDA). The GMP-based product qualification is mandatory for all firms that manufacture, package, label, or hold dietary supplements in the United States [7,15,31]. While statements of functional claims can be displayed on the packaging or labels according to the DSHEA, the manufacturing process is controlled by the GMP. 

**Figure 6:**
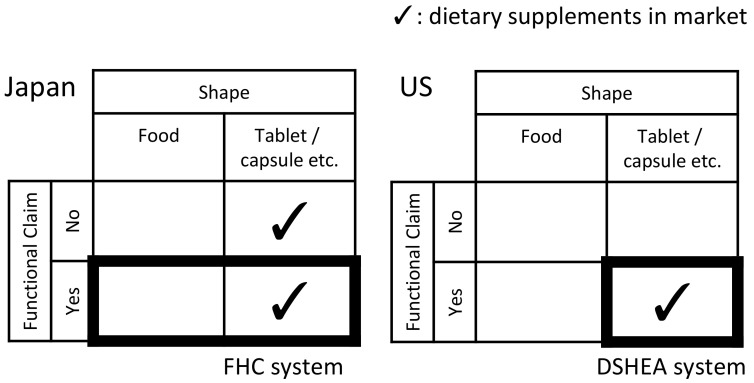
Comparison of legislative frameworks of functional foods in Japan and the United States. HC and DSHEA stand for Foods with Health Claim and Dietary Supplement Health Education Act, respectively.

On the other hand, Japan introduced a legislative framework for FFC in 2015. This system spans food and dietary supplements. However, dietary supplements under this system account for a small part of the Japanese dietary supplement market. In Japan, many dietary supplements are excluded from the system, and no legal system exists to comprehensively regulate dietary supplements. The relationship between dietary supplements and related legal systems is shown in Figure 6. Complex Japanese institutions are attributed to the historical path-dependency of a repeatedly revised system. It is difficult to redesign the Japanese legislative framework and legally define dietary supplements, because of the historical path-dependence of the system for dietary supplements and the market structure, which is dominated by dietary supplements outside the system, as mentioned. These factors developed a voluntary system without GMP being mandated in Japan. 

In Japan, the introduction of GMP was discussed since the 2000s. In 2005, MHLW required that the industry work on ensuring the safety of dietary supplements. MHLW published the “GMP guidelines”, specifying the GMP management process from the acceptance of raw materials to packaging and shipment of final products. In response, two organizations (JANFA and JIHFS) implemented voluntary GMP certifications. In 2008, a third party-certification system related to GMP was proposed in a report by the MHLW on the safety assurance of “Health Food”. Thereafter, the Health Food Certification Council was founded in 2009, and JANFA and JIHFS were designated as third party-certification organizations in 2014. Under the FFC system in 2015, GMP for dietary supplements was further promoted. According to the guidelines for notification for FFC by the CAA, it is strongly recommended that dietary supplements as FFC are manufactured at plants with GMP certification. Because one of the goals of FFC is safety assurance, manufacturers are required to ensure an appropriate manufacturing process. As the market for FFC is expanding, the GMP adoption rate will increase further.

According to the results of this study, food-based SMEs and companies with fewer products have lower rates of GMP adoption. Since these companies tend to deal with dietary supplements as a means of promoting regional economy and innovation, mitigating such trade-offs could be a key point of discussion to make the regulation more effective. Large-scale pharmaceutical manufacturers have the capability of pharmaceuticals. However, in Japan, because the preventive approach is not built into the healthcare system, few large-scale pharmaceutical companies deal with dietary supplements. In an effort to achieve disease prevention and health promotion endorsed by the central and local governments, the design and construction of an industrial/social system that integrates prevention and treatment would be effective to join more pharmaceutical companies.

As for the limitations of the present study, it employed cross-sectional data of 90 CMOs in Japan. As such, it was limited to a specific period and/or regional context. To obtain a deeper understanding of the adoption process of GMP for dietary supplements, it is necessary to conduct a time-course observation and analysis of these cases. In addition, some dietary supplement manufacturers keep the manufacturing process inside their own facilities and highly comply with GMP. Factors regarding the adoption of GMP in these companies would differ from those regarding the CMOs examined in this study. Further studies are needed in the future to address these points.

## 5. Conclusions

The present study focused on key success factors related to the adoption and implementation of GMP for dietary supplements in Japan. As a result of the empirical observations of 90 CMOs that are OEM manufacturers of dietary supplements, three factors were identified as affecting the adoption of GMP: company size, manufacturing capability of pharmaceutical products, and the number of categories of manufactured dietary supplements. Among those, expertise in manufacturing pharmaceutical products seemed to be the most influential in the dissemination and implementation of quality standards in the regional dietary supplement industry. These results and suggestions are expected to form a theoretical base for policymakers and regulatory authorities to reconsider the current regulatory framework and need for international harmonization, as well as to provide a cue for practitioners in industry on how to improve their capability to manufacture dietary supplements.

## Figures and Tables

**Table 1:** Descriptive statistics of the samples (90 original equipment manufacturers (OEMs) in the Japanese dietary supplement industry).

Variables	Mean	Standard Deviation	Maximum Value	Minimum Value
Compliance of GMP	0.51	0.50	1	0
Revenue (billion yen)	5.99	19.13	170.46	0.04
Number of employees	160.9	291.4	1837	3
License for manufacturing pharmaceutical products	0.27	0.44	1	0
License for manufacturing food products	0.51	0.50	1	0
Number of product categories	1.81	1.41	5	0

**Table 2:** Correlation coefficients of selected variables (revenue size, possession of a license for manufacturing pharmaceutical products, and the number of categories of manufactured dietary supplement products).

Variable	GMP	Revenue	No. of Employees	Pharmaceutical Dummy	Food Dummy
Revenue	0.231 *				
Number of employees	0.374 **	0.824 **			
Pharmaceutical manufacturer dummy	0.389 **	−0.002	0.098		
Food manufacturer dummy	0.066	−0.112	−0.096	0.188	
Number of product categories	0.546 **	0.121	0.317	0.169	0.121

*: *p* < 0.05; **: *p* < 0.01.

**Table 3:** Binomial logistic regression analysis of the adoption rate of GMP for dietary supplements with four independent variables.

Variables	Coefficien *t*	Odds Ratio	95% Confidence Interval		*p*-Value
			Lower	Upper	
Constant	−3.86	0.021	0.0025	0.106	0.00004 ***
Revenue size	0.04	1.04	1.01	1.09	0.019 *
Pharmaceutical manufacturer dummy	2.62	13.7	2.85	90.8	0.003 **
Food manufacturer dummy	−0.13	0.88	0.24	3.10	0.84
Number of product categories	1.37	3.93	2.14	8.61	0.00009 ***

*: *p* < 0.05; **: *p* < 0.01; ***: *p* < 0.001.
